# Exploring the Determinants of Suicidal Behavior: Conventional and Emergent Risk (DISCOVER): a feasibility study

**DOI:** 10.1186/s40814-015-0012-4

**Published:** 2015-05-09

**Authors:** Zainab Samaan, Monica Bawor, Brittany B. Dennis, Wala El-Sheikh, Jane DeJesus, Sumathy Rangarajan, Judith Vair, Heather Sholer, Nicole Hutchinson, Elizabeth Iordan, Pam Mackie, Shofiqul Islam, Mahshid Deghan, Jennifer Brasch, Lehana Thabane

**Affiliations:** 1 Department of Psychiatry and Behavioural Neurosciences, McMaster University, Hamilton, ON Canada; 2 Department of Clinical Epidemiology and Biostatistics, McMaster University, Hamilton, ON Canada; 3 Population Genomics Program, Chanchlani Research Centre, McMaster University, Hamilton, ON Canada; 4 MiNDS Neuroscience Program, McMaster University, Hamilton, ON Canada; 5 Population Health Research Institute, Hamilton Health Sciences/McMaster University, Hamilton, ON Canada; 6 St. Joseph’s Healthcare Hamilton, 100 West 5th Street, Hamilton, ON L8N 3K7 Canada; 7 Biostatistics Unit, Centre for Evaluation of Medicine, Hamilton, ON Canada; 8 System Linked Research Unit, Hamilton, ON Canada

**Keywords:** Pilot study, Suicide, Suicidal behavior, Risk factors, Biomarkers, Social determinants

## Abstract

**Background:**

Suicidal behavior is a growing public health concern resulting in morbidity and premature death. Although certain factors such as age, sex, and psychiatric disorders have been consistently reported to be associated with suicidal behavior, other factors including biological markers, diet, and physical activity may also influence suicidal behavior. The purpose of this pilot study was to evaluate the feasibility of conducting a full-scale study to identify the conventional and novel risk factors of suicidal behavior in individuals who made a recent suicide attempt.

**Methods:**

This pilot study was a case-control study of participants with recent (within 1 month of admission) suicide attempts admitted to hospital and compared to two control groups: 1) psychiatric inpatient participants without a history of suicide attempts and 2) community-based controls. We collected information on demographic variables, circumstances of suicide attempts (for cases), medical and psychiatric diagnoses, behavioral patterns, physical measurements, and social factors. Blood and urine samples were also collected for biological markers. Feasibility outcomes are as follows: 1) 50 % of all eligible cases will consent to participate, 2) 50 cases and 100 controls per year can be recruited, and 3) at least 80 % of the participants will provide blood samples for DNA and biological markers.

**Results:**

We recruited 179 participants in total; 51 cases, 57 psychiatric controls without suicide attempt, and 71 non-psychiatric controls in Hamilton, Ontario. Recruitment rate was 70 % (213/304), and we obtained urine and blood specimens from 90 % (191/213) of participants. Questionnaire completion rates were high, and data quality was very good with few data-related queries to resolve. We learned that cases tended to be hospitalized for long periods of time and the suicide attempt occurred more than a month ago in many of the cases; therefore, we expanded our inclusion criterion related to timing of suicide attempt to 3 months instead of 1 month.

**Conclusions:**

The study procedures needed certain modifications including extending the time between suicide attempt and date of recruitment, and more detailed questionnaires related to diet were necessary while other questionnaires such as social support needed to be shortened. Overall, this study showed that it is feasible to conduct a larger-scale study.

**Electronic supplementary material:**

The online version of this article (doi:10.1186/s40814-015-0012-4) contains supplementary material, which is available to authorized users.

## Background

Suicide is a leading cause of premature death worldwide, with over one million people dying by suicide each year [1]. Data from the World Health Organization shows that suicide accounts for 1.4 % of total deaths globally [2]. In Canada, the rate of completed suicide is estimated at 11.3 per 100,000 individuals with men three times more likely to die by suicide than women [2].

Suicidal behavior (SB) is a complex set of ideas, plans, and acts intended to end one’s life and is 10–20 times more common than completed suicide [3]. SB is reported to be among the most important risk factors for suicide [4-6]; however, it tends to be underreported due to several factors including a lack of recognition by services, denial of attempts, avoidance of legal inquiries, access to health services, stigma attached to suicidal behavior, financial consequences such as insurance claims, and lack of a national registry of SB. Over 90 % of individuals with SB have an identifiable psychiatric disorder [7]; however, not all patients with psychiatric disorders attempt suicide, suggesting a “diathesis” or predisposition to SB in susceptible individuals independent of underlying psychopathology [3].

Several demographic, biological, behavioral, and social factors influence SB, and it is likely that multiple other known and unknown factors contribute to SB. The interaction between environmental and biological factors is likely to provide a plausible understanding of the risk factors of suicide; however, this will require a refined phenotypic characterization of suicidal behavior and associated behaviors. The existing studies of SB provide valuable information on the conventional risk factors of suicide (such as age and sex); however, other risk factors are less investigated. For instance, many studies have sought to evaluate the links between diet and suicidal behavior. Increased suicide rates in people with low cholesterol or after lowering cholesterol through diet have been reported [8-13]. Studies of eicosapentaenoic acid (EPA), a polyunsaturated fatty acid found in fish oil, report a significant association between low EPA and SB [14]. Japanese studies reported lower risk of suicide in individuals consuming fish regularly [15]. Few studies have sought to determine the impact of exercise on suicidal behavior by demonstrating a direct link between exercise and suicide risk [16]. Thus, other dietary factors as well as exercise behaviors may also have a significant contribution to SB and are worth exploring further.

While several studies report risk factors associated with completed suicide [17], the risk factors associated with suicide attempts are likely to be different. In a study of genetic variants associated with suicide, suicide attempts were associated with a different genetic profile than completed suicide [18]. This suggests that SB is not a spectrum of severity phenomenon with suicide ideas on the milder end and completed suicide on the severe end, but likely to be better characterized as discrete categories with different risk factors for each category. Understanding both the inherent and modifiable risk factors of SB will assist in the prevention of SB and therefore completed suicide as well.

**Fig. 1:**
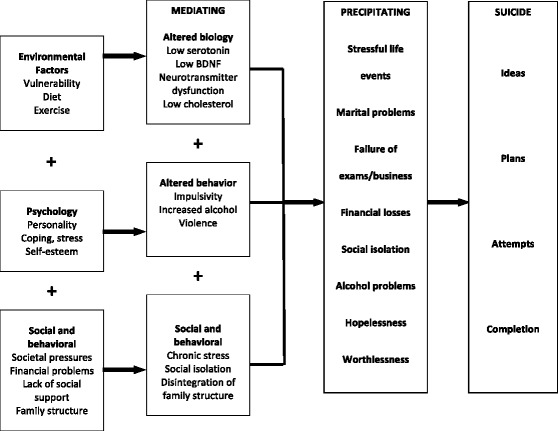
Suicidal behavior risk factors model

The main purpose of the Determinants of Suicide: Conventional and Emergent Risk (DISCOVER) study is to understand what risk factors are associated with suicide attempts that required hospitalization. We aimed to provide a comprehensive evaluation and phenotypic characterization of individuals with suicide attempts, and we propose a theoretical model of risk factors (Fig. 1) and how the interaction of these factors can lead to SB. We aim to systematically investigate conventional risk factors (including demographic variables, presence of chronic illness, socioeconomic factors, family history of suicide, and previous hospitalization) [19-21] as well as promising emergent risk factors (biological markers including peripheral levels of neurotransmitters [22, 23], chemokines [24], and genetic variants [18]; diet [25, 26]; vitamin D deficiency [27]; smoking [28]; and physical activity [29]) by designing a case-control study of individuals who made a recent (within 1 month of admission) suicide attempt and comparing them to two types of controls, psychiatric patients admitted to hospital without history of suicide attempts and non-psychiatric community-based controls.

The principal objective of this pilot study is to assess the overall feasibility of the main DISCOVER study by evaluating the following outcomes: 1) recruitment rate, 2) adherence to study procedures including completion of questionnaires and fasting for blood and urine samples, 3) feasibility of genetic and laboratory data collection, 4) preliminary evidence on emergent risk factors, and 5) assessment of overall study procedures including eligibility criteria.

## Methods

Eligible participants were recruited for the pilot study between March 2011 and December 2012 in Hamilton, Ontario, a mid-size Canadian city. The main aim of this pilot and feasibility study was to enhance the likelihood of success of the full-scale study. The objectives of this study are grouped into three main domains after Thabane and colleagues [30]: 1) to assess the feasibility of the study processes including the recruitment rate, refusal rate, adherence to overnight fasting procedure, eligibility criteria, understanding of study questionnaires; and 2) to establish resources needed as well as assess any management-related issues such as access to a private interview room when needed, and data management challenges such as missing variables and data variability. In addition we wanted to explore preliminary evidence for biological markers of suicide attempts.

We performed a case-control study of hospitalized individuals who attempted suicide within the past month and compared them to two control groups; one group consisting of hospitalized individuals with psychiatric disorders who did not have any history of suicide attempts, and another group consisting of individuals attending the general hospital for minor medical procedures, admitted to the hospital for medical/surgical causes, attending outpatient clinics, or community-based and not seeking health care.

### Criteria for assessing feasibility

We used the following parameters to assess the success of the feasibility study: 1) 50 % of all eligible cases consent to participate, 2) 50 cases and 100 controls per year can be recruited, and 3) at least 80 % of the participants will provide blood samples for DNA and biological markers.

### Setting and participants

The inclusion criteria for the pilot study were individuals aged 18 years or older who were able to provide written informed consent, communicate in English, and follow study procedures. The cases were individuals who made a recent serious (within past month) suicide attempt, which we defined as a suicide attempt that required medical/psychiatric intervention and admission to psychiatric or medical wards, and were inpatients at the time of study enrolment. To be defined as a case, patients were required to indicate they intended to die as a result of their self-harm. The cases were age (within 5 years) and sex matched to two controls. Control group 1 (control 1) participants were eligible for the study if they had psychiatric disorders without current or past history of suicide attempts and were admitted to the psychiatric hospital within the same time frame as the cases. Control group 2 (control 2) participants were recruited from the general population in the community or general hospitals and were included in the study if they did not have any history of suicide attempts.

Exclusion criteria include inability to provide informed consent or to follow study procedures. Given the heterogeneous nature of suicidal behavior, we defined a case as a person who made a suicide attempt that required hospital admission or who was considered based on clinical assessment at the time of presentation to have made a suicide attempt requiring hospital treatment. Potential study participants were approached by trained research personnel and detailed information about the study was provided. Individuals who agreed to participate in the study were asked to sign written informed consent. Each participant was interviewed using a structured interview with validated rating scales and questionnaires. All individuals underwent a psychiatric diagnostic interview and physical measurements, and blood and urine samples were obtained after an overnight fast. The pilot study was approved by the Hamilton Health Sciences (#10-661) and St. Joseph’s Healthcare (#11-3479) Research Ethics Boards.

Researchers for the inpatient recruitment approached the clinical staff and asked about suitable participants based on the study inclusion criteria. Clinical staff identified suitable patients after obtaining verbal consent from patients to be approached by research staff. Research staff would then obtain patient consent for participants. Upon obtaining consent, patients were asked questions pertaining to suicide attempt history, and medical records were used to confirm patients’ response.

For community recruitment, the researchers distributed advertisements in hospitals and community requesting volunteers to participate in the study. Upon obtaining consent, the researcher would take a history of suicide attempt and mental health from community participants. All participants were subject to screening and diagnostic interviewing for psychiatric disorders using the M.I.N.I. diagnostic assessment. Medical history was also obtained from all patients, including history of attending clinical services and current medications. The medical charts of included participants attending medical or surgical services were also checked for confirmation of diagnoses and history.

### Data collection and instruments

The DISCOVER study questionnaires were designed to acquire information on participants’ risk factors for suicide in a face-to-face interview format. We obtained data on socio-demographic variables (age, sex, ethnicity, religion, marital status, education, employment and current social support), medical history and current medications, diet, impulsivity, psychopathology, suicidal behavior (number of past attempts, details of methods used, age at first attempt, and family history of suicide), and physical measurements (blood pressure, pulse rate, height, weight, hip and waist circumference). The study questionnaires were compiled utilizing previously validated diagnostic and assessment tools including: Beck Suicide Intent Scale [31], M.I.N.I. International Neuropsychiatric Interview (Version 6.0) [32], Barratt Impulsivity Scale [33], Social Support Questionnaire [34], Borderline Symptom List 23 [35], Physical Activity Questionnaire [36, 37], and a short Food Frequency Questionnaire (FFQ) [38]. The FFQ consists of a food list, pre-determined portion size, and frequency of intake.

The study questionnaires were administered in hospital or community by trained research staff and subsequently entered into a confidential database at the project management centre of the Population Health Research Institute (PHRI). Fasting blood and urine samples were also obtained. There was no consultation with patients during the design of study. However, nurses provided feedback during study meetings on the processes, case report forms, as well as time it took to recruit and interview patients. Specific attention was paid to any feedback on patients’ comments during the interview, particularly whether patients found them long, tiring, and whether any questions were difficult to understand. Revisions were made to our questionnaires based on this feedback. For example, we changed the wording on one of our diet questionnaires from “rather much” to “often”.

We adhered to the standard consenting procedures and ethical practices outlined in the Declaration of Helsinki. During the consenting process, patients were informed of the study purpose, procedures, potential benefits, possible harms, as well as their ability to withdraw at any time. Participants were also required to consent to the collection and storage of biological material (blood samples). Regardless of reason, interviews were stopped at any times patients wished to withdraw.

Patients approached to participate could be voluntary (informal) or admitted under a form using the Canadian mental health act (formal). The Mental Health Act (MHA) in our jurisdiction does not automatically consider patients that were admitted informally to provide consent. In our system, patients can be admitted formally and still be considered competent to consent to treatment. Therefore, the patients included in this study contained a mixture of both informal and formal admissions; however, all patients are deemed competent to consent. Patients who are considered incompetent to consent to treatment are assessed using the MHA and would have a substitute decision maker to make decisions on their behalf. We did not approach patients who were deemed incompetent to consent. People who are assessed using MHA considered incompetent are usually patients with confusion, cognitive impairment, or severe psychotic symptoms impairing judgment, and therefore considered unable to make informed decisions.

### Statistical analysis

For the pilot study, we provide descriptive statistics expressed as mean and standard deviation (SD) for continuous variables and number (percent) for categorical variables. We used the Analysis of Variance (ANOVA) technique to compare means across different subgroups and *χ*^2^ test to compare proportions for categorical variables. Results from the ANOVA may suggest a difference exists between groups; however, we are unable to determine which groups are contributing to the difference. To circumvent this problem, we also performed a pairwise comparison and we reported the mean differences between cases versus control group 1 and cases versus control group 2. We have included these additional analyses in a supplementary appendix.

### Laboratory methods

Blood samples were collected, and after 30 min of clotting time, tubes were spun at 1500 × *g* (3000 rpm) for 15 min until blood was well separated (with the exception of the PAXgene tube). Plasma and serum samples were then aliquoted and stored in cryovials at the data collection site within 2 h of collection, then frozen in liquid nitrogen (−196 °C) at the Clinical Research and Clinical Trials Laboratory, Hamilton. Urine samples were also aliquoted and stored in liquid nitrogen (−196 °C) at the Clinical Research and Clinical Trials Laboratory for future analyses.

## Results

### Feasibility of the study processes

**Fig. 2:**
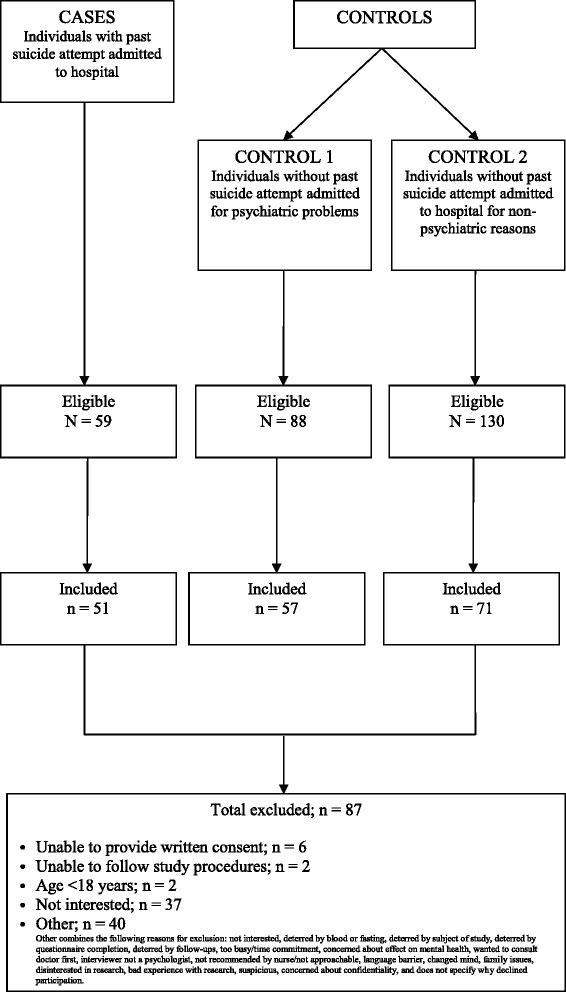
Flow diagram for participants included in study

We recruited participants from multiple sources (psychiatric hospital, general hospital, clinics and community) in Hamilton, Ontario, Canada, over a 20-month period from March 2011 to December 2012. In total, 213 participants were recruited (see Fig. 2 for participant flow diagram).

During the pilot study, we approached 304 individuals and enrolled 213 (70 %). Of the individuals enrolled in the study, 51 subjects had made a recent serious suicide attempt, 57 participants were in control group 1, 71 were in control group 2, and 34 individuals were individuals with past history of suicide attempts (this group was not included in the pilot study data analyses). The characteristics of the study participants are provided in Table 1, where we also present the differences between three groups of participants (cases, control 1, and control 2) using ANOVA. The mean differences between cases versus control group 1 and cases versus control group 2 can be found in Additional file 1: Table S1 and Additional file 1: Table S2 of the supplementary appendix.

**Table 1:** Characteristics of study participants (*n* = 179)

Variable	Case (*n* = 51)	Control 1 (*n* = 57)	Control 2 (*n* = 71)	*P* (ANOVA for mean difference, *χ*^2^ for categorical variables)
Age in years; mean (SD)	43.2 (13.3)	45.3 (14.0)	46.7 (18.7)	0.529
Female; *n* (%)	24 (47.1)	29 (50.9)	34 (47.9)	0.913
BMI (kg/m^2^); mean (SD)	28.3 (8.5)	28.7 (5.7)	28.3 (6.5)	0.097
Never married; *n* (%)	19 (37.3)	23 (40.4)	22 (31.0)	0.028
Completed college/university; *n* (%)	20 (37.3)	21 (36.8)	41 (57.7)	0.017
Currently employed; *n* (%)	12 (23.5)	19 (33.9)	39 (54.9)	0.001
Annual income <30 k/year; *n* (%)	20 (39.2)	21 (36.8)	12 (16.9)	0.051
Currently smoking; *n* (%)	25 (49.0)	18 (31.6)	10 (14.1)	<0.001
Family history of suicidal behavior; *n* (%)	20 (39.2)	16 (28.1)	17 (23.9)	0.081

*BMI* body mass index, *ANOVA* analysis of variance

### Establishing resources needed and assessment of study management

We were able to recruit 213 participants during a 20-month period, equivalent to a 70 % recruitment rate (304 potential participants approached and 213 participants recruited), demonstrating one of the feasibility outcomes for the full-scale study. Most participants (191 out of 213 participants (90 %)) provided early morning (between 7:00–9:00 am) fasting blood samples according to the study protocol, which ensures that biological testing will be feasible. Completion rates for the questionnaires were very high (>90 %), which suggests that our data collection and interview process are feasible to obtain and complete all the necessary information for the study (Table 2).

**Table 2:** Criteria for success of feasibility and feasibility outcomes

Feasibility outcome	Criterion	Results
Participant consent	50 % of all eligible cases consent to participate	70 % recruitment rate (213 participants out of 304 approached)
Number of participants recruited	50 cases and 100 controls per year can be recruited	51 cases and 128 controls recruited
Collection of blood samples	80 % of the participants will provide blood samples	90 % provided blood samples (191 out of 213 participants)

There were few reasons patients did not provide blood samples: participants did not return to the appointment to provide samples, they did not have the time to return, they were discharged, or we were unable to obtain their blood.

Research staff reported some difficulties when administering the full questionnaire, particularly the length of the interview process. Both interviewers and participants reported being unable to stay focused for the duration of the interview, or being interrupted by scheduled tasks for hospitalized participants.

### Preliminary results

The findings of this pilot study demonstrate that demographic factors including marital status, education level, employment status, and current smoking differed significantly between cases and controls (Table 1). The percentage of employed participants was lower among cases compared to both controls (unadjusted *χ*^2^ = 13.3, df = 2, *p* = 0.001) and the percentage of current smokers was significantly greater among cases compared to control groups (unadjusted *χ*^2^ = 22.6, df = 4, *p* < 0.001).

**Fig. 3:**
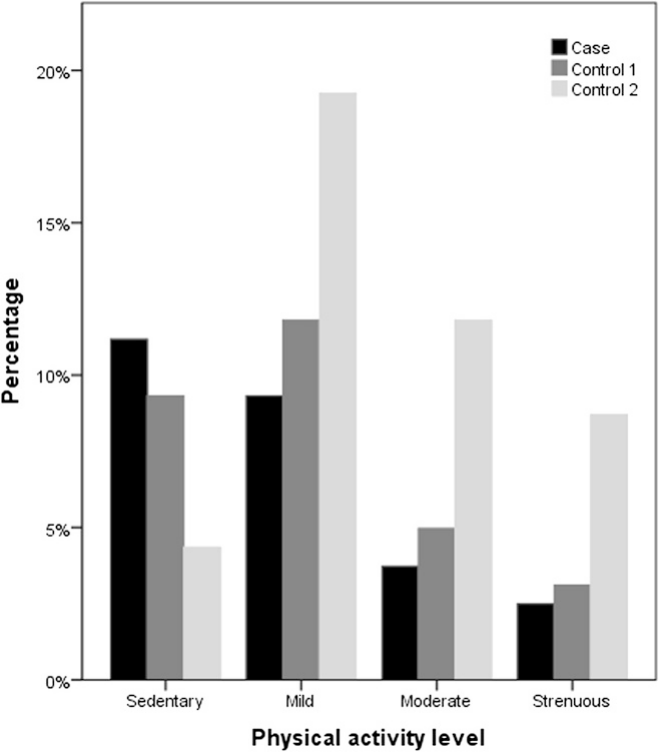
Physical activity in cases and controls

Preliminary results of the physical activity and leisure patterns for the past 12 months from the pilot data are shown in Fig. 3. Interesting trends are seen with cases being mostly sedentary compared to controls (unadjusted *χ*^2^ = 18.1, df = 6, *p* = 0.006).

**Fig. 4:**
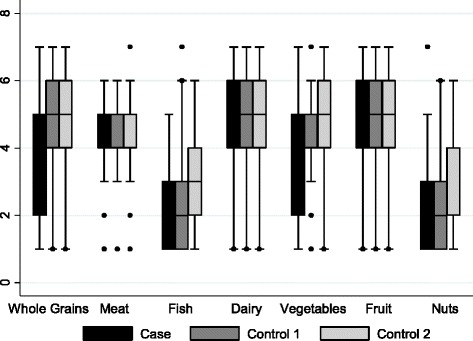
Mean number of servings of food groups by cases and controls. Legend for Fig. 4: The *error lines* represent variance around the mean estimates for number of food servings. These lines extend to the outer fences of interquartile range, which is represented by the size of the box. Any *dots* in this image represent outlier observations

We looked at dietary patterns for the past 12 months and our preliminary results showed that cases consumed less whole grains, vegetables, and fruits per day and less fish and nuts per week than controls (Fig. 4). Significant group differences between cases and controls were found for nut consumption per week (*F*(2,158) = 6.07, *p* = 0.003) and a trending pattern was found for raw vegetable consumption (*F*(2,158) = 3.00, *p* = 0.052). The error lines in this figure represent variance around the mean estimates for number of food servings. These lines extend to the outer fences of interquartile range, which is represented by the size of the box. Any dots in this image represent outlier observations.

## Discussion

The existing literature on risk factors of SB has identified multiple conventional risk factors for suicide; however, these studies are limited by modest sample size, inadequate characterization of the sample, and the inclusion of individuals with life-time history of suicidal attempts (retrospective data). Several studies lacked a control group and studies report conflicting findings. In addition, several studies adopted a nested case control design using convenience samples and investigated suicide attempts in a specific population based on psychopathology or other denominators. We aim to address these limitations with the design of the DISCOVER study; however, we opted to complete a pilot and feasibility study to enhance the likelihood of success of the main study.

This DISCOVER pilot study demonstrated feasibility to conduct the main study across many aspects including recruitment, participants’ willingness to provide blood and urine samples, questionnaire completeness, and encouraging data trends including confirmation of previously known risk factors. We would like to emphasize that the analyses presented in this study are strictly exploratory. The reporting of *p* values and any other significant results should be interpreted with caution.

### Key learning points from the pilot experience

Our study aims to investigate several risk factors for suicidal behavior; therefore, the number of assessments required to collect the information we need is extensive. We found that administering the full questionnaire was time-consuming for the interviewer and in some instances participants were unable to stay focused for the duration of the interview or had other scheduled tasks to attend to. Therefore, we modified the study procedures and conducted the interview over two sessions or as requested at the convenience of the participants.

During the course of the pilot recruitment, we found that many participants have been in the hospital under close supervision for a long duration prior to the study enrolment and therefore were not eligible to participate in the study due to timing of the 1-month inclusion criterion. This led us to revise our inclusion criterion of recent suicide attempt to include the 3 months prior to the study enrolment; however, potential cases would have remained in hospital at the time of study recruitment.

We learned that participants were having difficulty answering questions from the Borderline Symptom List because of the response options provided (i.e., not at all, a little, rather, much, very strong). Therefore, we provided alternative wording and explanations of the Likert scale. The Social Support Questionnaire seemed to be repetitive as participants generally reported one consistent source of support, and therefore, we will ask about the main source of support and avoid repeating the questions to participants.

### Study strengths and limitations

The study design we have chosen to investigate suicidal behavior risk factors is a case-control design where risk factors are compared among cases and controls. Ideally a cohort study design will be chosen to investigate risk factors; however, for the suicide risk factors study, the case control design is the most suitable based on the relatively low incidence of the suicidal behavior in the population (110/100,000 for suicide attempts). We would also need very large sample size and prolonged follow-up duration to acquire adequate number of cases, making a cohort design prohibitive to achieve. We believe that the case-control study design is the most efficient way to provide reliable information on the importance of several suicide risk factors when potential biases and confounders are minimized. By implementing a case-control study design, we have kept costs at a minimum and also reduced the possibility of losing participants to follow-up, which would be problematic with other study designs. We have also chosen to include two control groups so that more reliable comparisons can be drawn. However, the case-control study design is not without limitations. The results of any case-control study may be influenced by bias. In the case of social desirability, cases may under-report adverse lifestyle behaviors compared to controls or over-report advantageous lifestyle behaviors.

### Future directions

Recruitment for the full-scale DISCOVER study is underway, and we have incorporated several changes based on the results of this pilot study. First, we have modified the questionnaire according to our findings about the Borderline Symptom List, the Social Support Questionnaire, and the overall length of the interview. We also decided to stop collecting blood pressure and pulse rate as these measures are not risk factors or confounding factors for suicide attempt. After our preliminary results showed interesting results for the food patterns between cases and controls, we administered a comprehensive Food Frequency Questionnaire to measure participant habitual intake.

For the full-scale study, our ultimate goal is to conduct a prospective case-control study. We aim to include 100 individuals with recent suicide attempts requiring hospitalization and 200 controls with a yearly follow up of 5 years to record any new suicidal behavior events. The sample size for the study was conservatively estimated based on the recruitment rate and the relatively rare event of suicidal behavior requiring hospitalization.

## Conclusions

This pilot study examined the feasibility of the DISCOVER full-scale study, and we are able to conclude that it is feasible to conduct the full study with some modifications.

## Additional file

Additional file 1: Table S1. Comparative Demographic Characteristics Between Cases and Control Group 1. **Table S2** Comparative Demographic Characteristics Between Cases and Control Group 2.
