# Supplementary material for: Exploring the Determinants of Suicidal Behavior: Conventional and Emergent Risk (DISCOVER): a feasibility study
Source: Pilot Feasibility Stud. 2015 May 9;1:17. doi: 10.1186/s40814-015-0012-4 (PMC5154080; doi:10.1186/s40814-015-0012-4)
Supplement: Additional file 1: Table S1. — Comparative Demographic Characteristics Between Cases and Control Group 1. Table S2 Comparative Demographic Characteristics Between Cases and Control Group 2. [file 40814_2015_12_MOESM1_ESM.pdf]

Supplementary Appendix

Table S1: Comparative Demographic Characteristics Between Cases and Control Group 1

**Variable**	**Case (n = 51)**	**Control 1 (n = 57)**	**Effect**	
**Continuous variables**			**MD (95%CI)**	**P-value**
Age in years; mean (SD)	43.2 (13.3)	45.3 (14.0)	-2.10 (-7.32 to 3.12)	0.427
BMI (kg/m^2); mean (SD)	28.3 (8.5)	28.7 (5.7)	-0.40 (-3.13 to 2.34)	0.772
**Binary variables**	**Case (n = 51)**	**Control 1 (n = 57)**	**OR (95% CI)**	**P-value**
Female; n (%)	24 (47.1)	29 (50.9)	0.86 (0.38 to 1.96)	0.705
Never married; n (%)	19 (37.3)	23 (40.4)	0.88 (0.38 to 2.05)	0.844
Completed college/university; n (%)	20 (39.3)	21 (36.8)	1.11 (0.47 to 2.59)	0.844
Currently employed; n (%)	12 (23.5)	19 (33.9)	0.62 (0.24 to 1.56)	0.293
Annual income<30k/year; n (%)	20 (39.2)	21 (36.8)	1.06 (0.66 to 1.72)	0.844
Currently smoking; n (%)	25 (49.0)	18 (31.6)	2.08 (0.89 to 4.92)	0.078
Family history of suicidal behaviour; n (%)	20 (39.2)	16 (28.1)	1.65 (0.68 to 4.01)	0.229

Table S2: Comparative Demographic Characteristics Between Cases and Control Group 2

**Variable**	**Case (n = 51)**	**Control 2 (n = 71)**	**Effect**	**P**
**Continuous variables**			**MD (95%CI)**	**P-value**
Age in years; mean (SD)	43.2 (13.3)	46.7 (18.7)	-3.50 (-9.55 to 2.55)	0.255
BMI (kg/m^2); mean (SD)	28.3 (8.5)	28.3 (6.5)	0.00 (-2.69 to 2.69)	>0.999
**Binary variables**	**Case (n = 51)**	**Control 2 (n = 71)**	**OR (95% CI)**	**P-value**
Female; n (%)	24 (47.1)	34 (47.9)	0.97 (0.44 to 2.11)	>0.999
Never married; n (%)	19 (37.3)	22 (31.0)	1.32 (0.58 to 3.02)	0.561
Completed college/university; n (%)	20 (37.3)	41 (57.7)	0.47 (0.21 to 1.05)	0.066
Currently employed; n (%)	12 (23.5)	39 (54.9)	0.25 (0.10 to 0.60)	<0.001
Annual income<30k/year; n (%)	20 (39.2)	12 (16.9)	3.17 (1.27 to 8.06)	0.007
Currently smoking; n (%)	25 (49.0)	10 (14.1)	5.87 (2.29 to 15.53)	<0.001
Family history of suicidal behaviour; n (%)	20 (39.2)	17 (23.9)	2.05 (0.87 to 4.83)	0.072

Supplementary analyses were conducted using WINPEPI

Abramson, J.H. WINPEPI updated: computer programs for epidemiologists, and their teaching potential. Epidemiologic Perspectives & Innovations. 2011, 8:1
